# Comparing Prodrugs with Acyclovir for Treating Postherpetic Neuralgia among Herpes Zoster Patients: A Systematic Review and Meta-Analysis

**DOI:** 10.3390/healthcare10071181

**Published:** 2022-06-24

**Authors:** Chung-Hsin Yeh, Ko-Shih Chang, Sheng-Shiung Huang, Shiow-Luan Tsay, Jung-Mei Tsai, Ya-Jung Wang

**Affiliations:** 1 Department of Neurology, Yuan Rung Hospital, Changhua 510005, Taiwan; shium8852@gmail.com; 2 Department of Nursing, College of Nursing and Health, Da-Yeh University, Changhua 515006, Taiwan; sshuang@mail.dyu.edu.tw (S.-S.H.); jungmei56@gmail.com (J.-M.T.); wangyj@mail.dyu.edu.tw (Y.-J.W.); 3 Department of Cardiology, Yuan Rung Hospital, Changhua 510005, Taiwan; yuanrung96@gmail.com; 4 Department of Nursing, Mackay Memorial Hospital, Taipei 25160, Taiwan

**Keywords:** herpes zoster, postherpetic neuralgia, antiviral agents, acyclovir, meta-analysis

## Abstract

Postherpetic neuralgia (PHN) is a common, painful, and long-term complication of herpes zoster (HZ). PHN increases the demand for healthcare services and, previous studies showed that patients who received antiviral agents were less likely to develop PHN. The objective of this study was to compare the efficacy of prodrugs and acyclovir in treating PHN among patients with HZ. The search included the PubMed, Medline, Embase, and Cochrane Center of Register of Controlled Trails databases through February 2022. Clinical trials and randomized controlled trials (RCTs) involving antiviral agent intervention for HZ patients diagnosed with PHN were eligible for inclusion. A meta-analysis was conducted to calculate pooled risk ratios (RRs) with 95% confidence intervals (CIs) with a fix-effect model. Five RCTs with 1147 HZ patients met our eligibility criteria. Our meta-analysis found that there was a significantly lower risk of PHN for members of the prodrugs group (famciclovir and valaciclovir) compared with those who received acyclovir (RR = 0.86, 95%, CI: 0.75 to 0.98, *p* = 0.03). The review of studies indicated that the efficacy of prodrugs was better than acyclovir for reliving PHN.

## 1. Introduction

Herpes zoster (HZ), commonly known as shingles, results from the reactivation of the varicella-zoster virus (VZV). The symptoms include a painful, itchy, and tingly rash in one or two adjacent dermatomes that progresses topically to a vesicular-pustular appearance in 7 to 10 days, eventually evolving into a crust and resolving a few weeks after onset [1,2]. One recent systematic review indicated that the incidence rate of HZ has ranged from 5.23 to 10.9 per 1000 person years [3]. The incidence rates of HZ reported in other studies conducted in the United States (ranging from 4 to 7.2) [4,5], Canada (3 to 5) [6,7], United Kingdom (8.80) [8], Spain (8.29) [9], Germany (6.7) [10], China (6.64) [11], South Korea (1.87 to 5.1) [12,13], Japan (4.15 to 4.79) [14,15], and Taiwan (5.65) [16] revealed a trend of increasing incidence of HZ with increasing age, making the condition a major health burden on the elderly [17,18]. Thus, HZ has been a common clinical problem, with the elderly (defined in this context as those over 50 years of age) and immunocompromised patients again being especially susceptible [19,20]. According to the data from the National Development Council, Taiwan became an aged society (>14% of the population were elderly) in 2018 and will become a super-aged society (>20% of the population over the age of 64) in 2026 [21]. Therefore, HZ and its complications represent a critical health issue as the population in many countries continues to age.

HZ patients commonly suffer from postherpetic neuralgia (PHN), this being the most common and bothersome complication of the disease [2,17,22]. PHN is defined as pain that persists after the rash caused by HZ [23]. Cases of PHN are classified based on the amount of time that elapses between the onset of the rash and the persistence of the pain. Thus, persistent pain within 30 days after the onset of the rash is called acute herpetic neuralgia; within 30 to 120 days after the onset of the rash is called subacute herpetic neuralgia; and pain that persists more than 120 days after the onset of the rash is called PHN [24,25,26]. Many patients with HZ continue to suffer from moderate to severe pain after the rash has resolved [20,27], with their PHN limiting daily activities and quality of life for months to years afterward [20,28,29]. 

Specifically, PHN occurs in 10% to 34% of HZ patients and becomes more likely with age [6,22,30,31,32,33,34,35]. Lu et al. (2018) found that HZ patients consumed more of all types of healthcare services after the onset of PHN than individuals without HZ. Though the mechanism behind the pathophysiology of PHN remains unclear, studies have indicated that it may develop as a result of damage to peripheral and central neurons, possibly as a consequence of the immune response caused by the reactivation of the virus [36,37]. Zhu et al. (2009) further suggested that an inflammatory response such as IL-6 released may be associated with the development of PHN [38].

Though treatments for PHN are available—such as gabapentinoids, topical lidocaine, opioid analgesics, pregabalin, duloxetine, venlafaxine, certain opioids, and tricyclic antidepressants (TCAs) [39,40,41]—patients who received these medications in research studies found them to be either partly or wholly ineffective. There were also new drugs for treating HZ such as amenamevir and FV-100 in recent years. Amenamevir is an innovational and effective inhibitor of helicase–primase. Kawashima and colleagues (2017) conducted a multicenter, randomized, double-blind, phase 3 study to compare the efficacy and safety of amenamevir with valaciclovir in patients with HZ. Their results showed that amenamevir was more effective and well-tolerated than valaciclovir. However, there was no statistical difference in the incidence of PHN between amenamevir and valaciclovir [42]. Additionally, the FV-100 is a prodrug for the bicyclic nucleoside analog CF-1743 [43]. Previous RCT also indicated that FV-100 for the treatment of HZ could reduce the pain associated with HZ and the incidence of PHN [44]. Those drugs are very new, more research is needed to validate overall efficacy. 

In any case, it would be more efficient to prevent the development of PHN in the first place through the administration of antiviral agents than to treat the symptoms [45]. Furthermore, Advisory Committee on Immunization Practices (ACIP) suggested that receiving an HZ vaccine is recommended for the prevention of HZ and related complications for immunocompetent adults aged ≥ 50 years [46]. Both Zostavax (ZVL) and Shingrix (RZV) are approved for use in more than 35 countries, including the European Union, the United States, Canada, Japan, Australia, South Korea, Singapore, and China [47]. Previous studies also demonstrated that the risk of PHN is lower in people who experience HZ after vaccination than in unvaccinated people [35,48,49].

Additionally, the timing of the treatments for PHN may influence their effectiveness [50]. Specifically, it appears that antiviral agents are most effective in mitigating the impact of the reactivation of HZ when administered within 72 h after the onset of the rash [2,51]. Antiviral agents such as the aforementioned acyclovir (Zovirax), famciclovir (Famvir), and valaciclovir (Valtrex) have been the primary first-line treatment for HZ. Moreover, previous studies demonstrated the efficacy of antiviral medications in the treatment of HZ [52,53,54]. 

As mentioned before, antiviral agents may help to alleviate PHN [55,56,57,58,59,60]. Thus, for instance, valaciclovir and famciclovir have proved effective in relieving pain associated with HZ and PHN [60], as has topical 5% acyclovir administered in the early stages of the rash [61]. Acyclovir, which has been in use since the 1990s, was among the first antiviral agents to be widely administered in the treatment of HZ. Though this drug has the advantage of lower cost than treatment with prodrugs such as famciclovir and valaciclovir [2], the dosing schedule tends to be quite frequent and, therefore, inconvenient, with patients often receiving 800 mg orally five times per day for 7 days. The newer drugs, in contrast, are administered only three times per day for 7 days, with the doses of valaciclovir being greater than those of famciclovir (1000 mg and 500 mg, respectively) [2]. Therefore, while the dosing schedule for famciclovir and valaciclovir is simpler than that for acyclovir, all three drugs have proved effective in attenuating PHN [23]. The complicated dosing schedule, though, could result in patients’ non-adherence and, thus, adverse treatment outcomes [62]. 

Prodrugs such as valaciclovir and famciclovir have also been shown to accelerate the resolution of persistent pain associated with HZ and to help prevent PHN. For example, Beutner et al. (1995) reported that valaciclovir accelerated the resolution of pain associated with HZ (*p* < 0.05) and reduced the duration of PHN significantly better than acyclovir. Likewise, a randomized controlled trial (RCT) by Trying et al. (1995) found that patients who received famciclovir experienced more rapid resolution of their PHN than those who received a placebo (hazard ratio = 1.70, 95% CI: 1.10 to 2.70) [63], and, in another study, valaciclovir proved superior to acyclovir in easing both HZ-associated pain and PHN [57]. Other RCTs found famciclovir and valaciclovir to be of equivalent effectiveness in reducing the duration of PHN [64,65]. Additionally, previous RCTs have shown famciclovir to be not only as effective as acyclovir in ameliorating acute-phase pain but also well-tolerated and to have an advantageous adverse event profile [58,59]. Both valaciclovir and famciclovir, then, appear to be more convenient, effective, and safe than acyclovir for treating patients with HZ.

The evidence for the effectiveness of acyclovir in treating PHN, by contrast, has been inconsistent. For example, one meta-analysis of four double-blind RCTs found that this antiviral relieved pain and lower the proportion of patients with PHN at 3 and 6 months [66]. Morton and Thomson (1989) likewise found that acyclovir reduced the weekly prevalence of pain as well as the monthly prevalence of chronic pain [67], and Jackson et al. (1997) calculated that the summary pooled odds ratio of PHN 6 months after the onset of the rash to be 0.54 (95% CI: 0.36–0.81) [68]. McGill and White (1994) also reported that acyclovir could prevent the incidence of PHN [69]. However, other studies found that acyclovir did not prevent PHN [42,45,70,71,72,73,74,75,76,77,78,79,80,81,82]. Beyond antiviral agents, as mentioned, there have also been reports that tricyclic antidepressants, topical capsaicin, gabapentin, oxycodone, pregabalin, long-acting opioids, and tramadol alleviated PHN, though the long-term and clinical benefits of these treatments remain uncertain [28,83]. Additionally, as discussed, in the research that has shown antiviral agents to be effective in treating HZ, their specific impact on PHN has rarely been discussed. A recent study indicates that patients with HZ used healthcare more than those without the disease and the growing burden of PHN on healthcare systems have already been noted [16], as well as the association of this burden with the aging populations in many countries [6,30]. Accordingly, systematic reviews have identified HZ as a critical global health burden [3,32]. To date, though, there has been relatively little research comparing the efficacy of these antiviral agents in relieving PHN. The objective of this study was to compare the efficacy of newer antiviral agents’ prodrugs and acyclovir in treating PHN among patients with HZ.

## 2. Materials and Methods

This study took the form of a meta-analysis of RCTs that investigated the association between acyclovir and PHN. We conducted it in accordance with the Preferred Reporting Items for Systematic Review and Meta-analysis (PRISMA) protocol [64]. 

### 2.1. Evidence Search

We searched relevant studies from the PubMed, Medline, and Cochrane Central Register of Controlled Trails databases through February 2022. We relied on the PICO framework to identify potentially relevant studies in the search, including both the indexed terms and Medical Subject Headings (MeSH) terms found in the titles and/or abstracts. The specific search terms were Population (P): herpes zoster; Intervention (I): acyclovir; Comparison (C): placebo or prodrugs; Outcome (O): PHN.

### 2.2. Eligibility Criteria

Clinical studies and trials and RCTs involving acyclovir intervention for patients diagnosed with HZ were eligible for inclusion in this review. We compared the results of treatments with acyclovir and the prodrugs with placebos after the onset of HZ with PHN. The cut-off time for PHN varied across the studies. To include as many RCTs of the antiviral PHN treatments as possible in our sample, we used a cut-off time of one month after the onset of the rash (i.e., at least 30 days from the onset), which is consistent with the definition of acute PHN in the previous literature [24,25,26]. The further criteria, selected to avoid any misinterpretation of the data or other results, included limiting the sample to full-text articles written in English for which the data were available. The details of the search strategy are provided in Supplementary Table S1.

### 2.3. Data Collection and Risk Assessment

EndNote software version 20.0 (London, UK) served to remove the duplicate records from the studies that the search of the databases identified. Two of the authors (C.-H.Y. and K.-S.C.), working independently, assessed the risk of bias in the eligible studies and analyzed them. They identified potentially relevant research by examining the titles and abstracts and then extracted the data from and assessed the quality of each study. The other co-authors weighed in when there was uncertainty regarding the relevance of a study. 

### 2.4. Statistical Analysis

We conducted the meta-analysis using ReviewManager software (version 5.3, Nordic Cochrane Centre, Cochrane Collaboration (London, UK) according to the manufacturer’s guidelines. We also used this software to calculate the pooled risk ratios (RRs) with 95% confidence intervals (CIs) in a fixed-effect model across the eligible studies. Our examination of the heterogeneity of the studies determined the *I^2^* value to be greater than 50%, indicating that there was, in fact, substantial heterogeneity [74]. 

## 3. Results

### 3.1. Characteristics of the Relevant Studies

**Figure 1:**
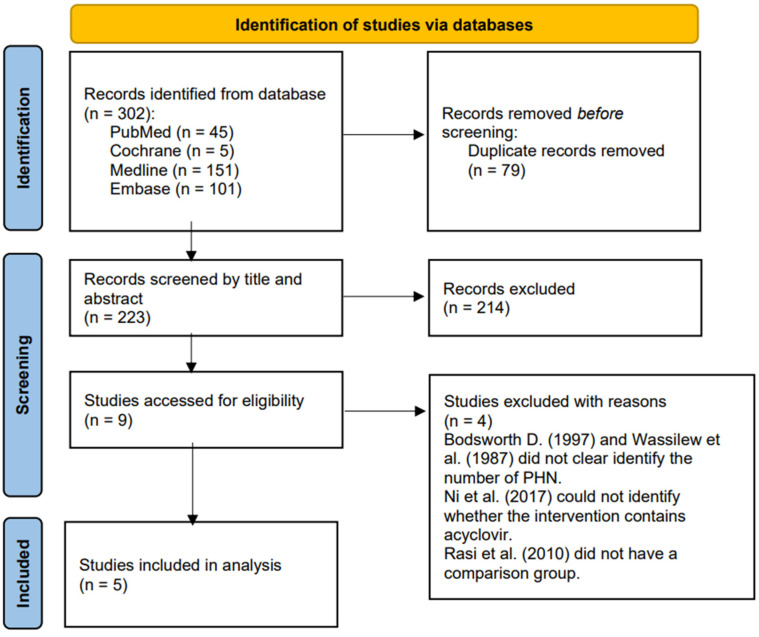
PRISMA 2020 flow diagram for the identification of relevant studies.

Figure 1 presents the process by which we identified 302 relevant studies in the four databases. After removing the duplicates, we screened 223 studies based on the titles and contents of the abstracts and excluded 214 that did not meet our inclusion criteria, leaving nine for further evaluation [43,52,53,67,75,76,77,78,79]. After examining the full texts of these articles, we excluded four clinical trials [50,76,77,80] because of a lack of a comparison group or of clarity regarding the number of PHN patients who received acyclovir. The five remaining studies included four double-blind RCTs [52,53,67,78] and one comparative randomized clinical study [80] that we subjected to further analysis. Supplementary Table S2 presents the characteristics of these five studies.

### 3.2. Relevant Studies

We found many studies of antiviral treatments for acute HZ, but only a few addressed relevant endpoints, such as the incidence and duration of PHN or the associated pain after the acute phase. The five studies that met our criteria for the meta-analysis included a total of 1147 participants. Regarding the inclusion and exclusion criteria, four of the studies recruited participants who had HZ presenting within 72 h after the onset of the rash [53,54,67,80]. Surman et al. (1990), who conducted the fifth study, also recruited patients with HZ but did not mention the time elapsed after the onset of the rash. Two of the studies looked at participants over 50 years of age [52,53], another two looked at participants over 40 [78,80], and one established the age cut-off at 16 years [59]. The sample size varied across the studies, ranging from 21 to 760, with three involving more than 100 participants [52,53,80]. All five studies reported the demographic characteristics of the participants, and three reported the absence of any statistically significant differences in the demographic characteristics of the intervention and control groups [52,53,67]. Surman et al. (1990) and Gopal et al. (2013) presented the distribution of gender and age but did not mention the statistical test used for comparison among the groups in terms of gender or age. Gopal et al. (2013) also noted that the acyclovir and famciclovir groups were comparable in terms of the time of screening and pain intensity. The studies excluded participants under the age of 18 (or, in one case, as discussed, 16), as discussed, as well as those who were pregnant, nursing, HIV seropositive, allergic to acyclovir, receiving chemotherapy or other antiviral treatments, or suffering from immunosuppression, hepatic or renal dysfunction, allergies, dementia, psychosis, or severe complications of HZ [52,53,67,78,80]. Surman et al. (1990) further excluded patients suffering from a congenital disease, acquired or steroid-induced immunodeficiency, impaired hepatic function, or gastrointestinal dysfunction. None of the five studies included immunosuppressed participants.

To evaluate the effects of acyclovir on the treatment of HZ and PHN, four of the studies used a dose of 800 mg five times daily while Surman et al. (1990) used this dose six times daily during waking hours for 12 weeks [80]. Regarding the control group, three of the studies administered a placebo to some of the participants [53,67,78]. For the control groups in the other studies, Beutner et al. (1995) used valaciclovir at a dose of 1000 mg, and Gopal et al. (2013) used famciclovir at a dose of 750 mg, in both cases 3 times daily for 7 days.

All of the studies reported the outcome measurement. Huff et al. (1988) and Morton and Thomson (1989) reported the prevalence of the pain experienced by the participants and clinically defined PHN as appearing one month after the onset of the acute herpetic rash. Surman et al. (1990) used the McGill Pain Questionnaire [81] to assess the presence and severity of the pain and clinical improvement to identify patients with PHN but did not specify the cut-off time for it. Gopal et al. (2013) used the visual analog scale to evaluate the duration and intensity of the pain for 6 weeks and reported the cases of PHN in each group but provided no clear definition of it. Beutner et al. (1995) evaluated pain based on the participants’ daily records (over 30 days) and assessed its severity weekly (over 24 weeks), reporting the incidence of PHN, again offering no definition for it. 

Three of the studies described the severity of pain using various pain evaluation methods over the various periods of study. Surman et al. (1990) employed the pain rating index (PRI) and Present Pain Index (PPI) from the McGill Pain Questionnaire [81] to evaluate the status of the participants’ pain over the six months after their initial evaluations. Those who received acyclovir (mean ranging from 15.83 to 21.11) reported lower PRI values than those who received the placebo (mean from 12.50 to 285.60). Conversely, the participants who received acyclovir (mean from 2.09 to 2.73) reported higher PRI values than those who received the placebo (mean from 1.17 to 2.30). Additionally, the acyclovir group demonstrated significantly higher PRI values from 2 weeks to 3 months (F values ranging from 13.29 to 6.31, *p* < 0.05) and PPI values at 3 months (F = 7.88, *p* < 0.05). Beutner et al. (1995) assessed the severity of the pain with the Gracely Pain Intensity Scale over 24 weeks and found no treatment-related trends across all of the participants, though those who received valaciclovir experienced significantly more rapid resolution of their pain than those who did not (*p* = 0.03). Gopal et al. (2013) employed a visual analog scale to assess the level of pain (ranging from 0 = no pain to 9 = severe pain) for 6 weeks. Rather than reporting the values of scales in the acyclovir and famciclovir groups, they reported the level of pain, with both groups experiencing moderate pain at the time of screening. 

Four of the studies mentioned adverse events, the exception being Surman et al. (1990). The most common were nausea, headache, and dyspepsia. Beutner et al. (1995) reported that the participants in their study experienced vomiting, diarrhea, constipation, asthenia, dizziness, anorexia, and abdominal pain, and Gopal et al. (2013) reported that 7% of the participants in their study experienced constipation. None of the studies reported serious adverse events nor any significant differences between the treatment and control groups in this regard [52,53,67,80]. One participant died in each of the groups in the study by Morton and Thomson (1989), but neither death was related to the intervention. The adverse experience profiles of the two valaciclovir treatments were very similar to each other and to the profiles for the acyclovir treatment.

### 3.3. Excluded RCTs

Of the four RCTs that we excluded from the assessment of the eligible stage [50,76,77,79], Bodsworth et al. (1997) and Wassilew et al. (1987) did not state clearly the number of PHN patients who participated. Ni et al. (2017) administered as an intervention either a standard therapy (oral antivirals and analgesics) alone or a standard therapy plus subcutaneous injections of triamcinolone and lidocaine but did not state clearly the number of participants who received acyclovir alone. Lastly, the study Rasi et al. (2010) had no comparison group [77].

### 3.4. Risk of Bias in the Five Studies

**Figure 2:**
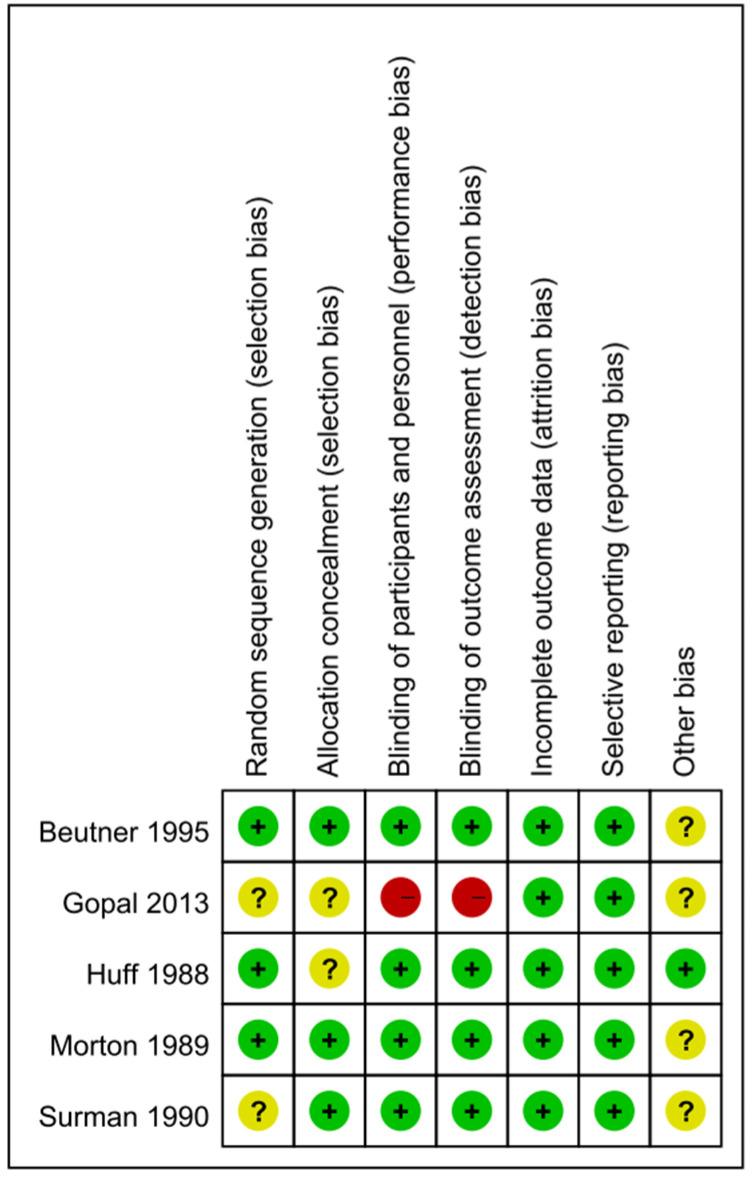
Summary of the assessment of the risk of bias.

Four of the five studies had a randomized double-blind design, the exception being that by Gopal et al., which had a comparative randomized clinical design [78,80]. Supplementary Table S3 and Figure 2 present the risk of bias in the five studies. Of the cells in the included studies, 22.86% were rated as having an uncertain risk of bias in the main dimensions of assessment of risk of bias (Figure 2). We rated only one study as having a high risk of bias in the blinding procedures [80]. The studies by Gopal et al. (2013) and Surman et al. (1990) involved a double-blind procedure, though these researchers did not specify their methods for generating the random sequences, and neither Gopal et al. (2013) nor Huff et al. (1988) specified their technique for allocation concealment. Surman et al. (1990) reported the number of participants who dropped out of the trial but without indicating the group or groups to which those who did so belonged. Among the potential biases in the other four studies, the participants in one were outpatients [52], three included participants who were receiving other treatments [67,80], and one included participants who continued taking their current medications [78]. We reasoned that the failure to exclude such participants may have resulted in underestimation of the effects of the interventions. Additionally, the participants in two of the studies were asked to report the development of PHN over the entire follow-up period [53,67]. Because Beutner et al. (1995) assessed pain based on the participants’ daily records, their results may have been subject to recall bias in the collection of data relating to adverse events, therefore introducing an error into the inferences drawn from the results.

### 3.5. Blinding

As has been seen, four of the studies involved double-blind procedures, which the researchers described well with respect to the participants, personnel, and outcome assessment [52,53,67,78]. Gopal et al. (2013), on other hand, did not explain their blinding procedure clearly [80].

### 3.6. Incomplete Outcome Data

All five studies reported the numbers of participants who dropped out and of those who completed the follow-ups. None dropped out of the study by Gopal et al. (2013).

### 3.7. Selective Reporting

Again, all five studies reported the results of their outcome assessments, having been designed primarily to assess the efficacy of the acyclovir intervention after the onset of HZ in relieving PHN. This being the case, the selective reporting bias was small. 

### 3.8. Other Potential Sources of Bias

**Figure 3:**
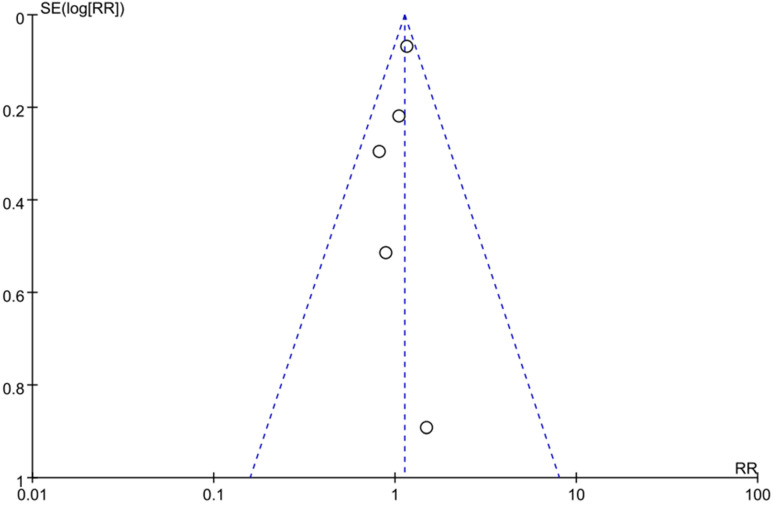
The funnel plot of current studies. The circle presents the risk ratio and standard error (SE) of the log RR of each study.

To assess the publication bias, we conducted a funnel plot, which showed an asymmetry pattern (Figure 3). In keeping with the recommendations for testing funnel plots in the Cochrane Handbook (Chapter 13), we kept the number of studies included in this meta-study small to distinguish chance asymmetry from real asymmetry and to make full-text evaluation feasible [81].

### 3.9. The Quality of the Evidence

We assessed the quality of the evidence based on the GRADE criteria [83]. At the beginning of the assessment, all of the RCTs were considered to have a high certainty of evidence. Only the study by Gopal et al. (2013) showed a high risk of bias—regarding the blinding procedures, as discussed—meaning that 22.86% of the cells of the included studies were rated as having an uncertain risk of bias and 6% of the cells were identified as having a high overall risk of bias (Figure 2), a situation that, in turn, downgraded the level of the evidence. A total of 1147 participants (Acyclovir group:462; Control group: 413) were analyzed among the five including RCTs. The main results indicated that the effect of acyclovir on the PHN among patients was statistically insignificant (RR = 1.13, 95% CI: 0.99 to 1.28, *p* = 0.06). Hence, we downgraded the level of evidence. We also downgraded the level of evidence since our study was, as discussed, restricted to English-language and full-text articles for which the data were available. As result, the certainty of the evidence of our study was low and the effect estimate was limited.

### 3.10. Effects of the Interventions

#### 3.10.1. Prodrugs Groups Versus Acyclovir Groups

**Figure 4:**
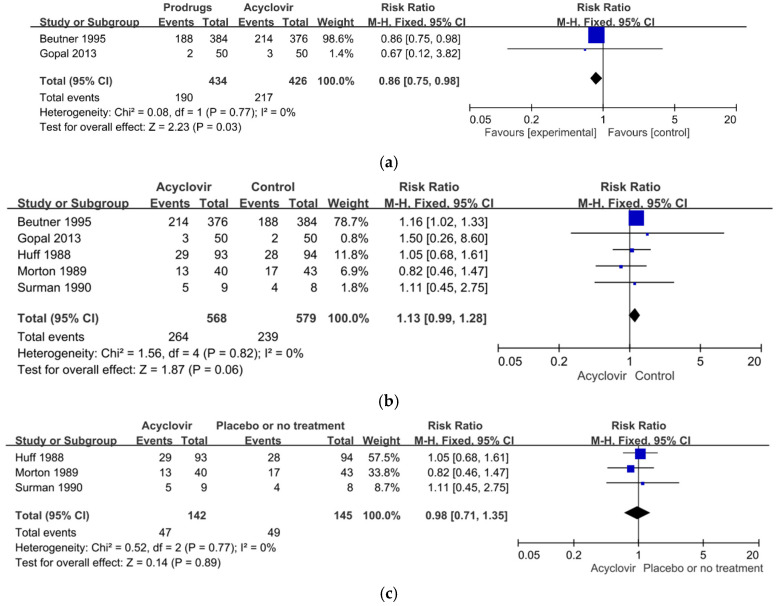

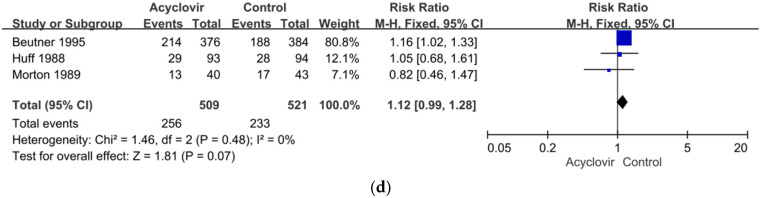
(**a**) Forest plot comparison: prodrugs versus acyclovir in relieving of PHN within one month of the onset of the acute herpetic rash. (**b**) Forest plot of comparison: acyclovir versus the control including the placebo or prodrugs in the relieving of PHN within one month after the onset of the acute herpetic rash. (**c**) Forest plot of comparison: acyclovir versus the placebo in the relieving of PHN in one month after the onset of the acute herpetic rash. (**d**) Sensitivity analysis: acyclovir versus the control group excluding the high-risk studies in relieving of PHN within one month of the onset of the acute herpetic rash.

Two studies, involving 860 participants in all, compared the effect of acyclovir with that of produgs (valaciclovir or famciclovir) [52,80]. The results of our meta-analysis showed that the RR for PHN events among the participants who received the prodrugs was significantly higher than among those who received acyclovir (RR = 0.86, 95%, CI: 0.75 to 0.98, *p* = 0.03). In other words, the prodrugs were significantly more effective than acyclovir in relieving PHN within one month after the onset of the rash. The results of a heterogeneity assessment showed no significant heterogeneity between these two studies (χ^2^ = 0.08, *p* = 0.77, *I*^2^ = 0%) (Figure 4a).

#### 3.10.2. Acyclovir Groups Compared with the Control Groups

The five studies, as discussed, compared the effects of acyclovir on the members of the treatment groups with the progress of PHN in the members of the control groups who received either a placebo or prodrugs (famciclovir or valaciclovir). Our meta-analysis showed that the incidence of PHN events among the members of the acyclovir groups was only slightly, and not significantly, higher than among the members of the control groups (RR = 1.13, 95% CI: 0.99 to 1.28, *p* = 0.06). In other words, acyclovir did not prevent PHN within one month after the onset of the rash. The results of a heterogeneity assessment showed no significant heterogeneity among the five studies (χ^2^ = 1.56, *p* = 0.82, *I*^2^ = 0%) (Figure 4b).

#### 3.10.3. Acyclovir Versus Placebo

Three of the studies, involving 287 participants in all, compared the effects of acyclovir with the administration of a placebo [53,67,78]. The results of our meta-analysis showed that the incidence of PHN events for the participants who received acyclovir was lower than for those who received the placebo or no treatment (RR = 0.98, 95%, CI: 0.71 to 1.35, *p* = 0.89), but the difference was not significant. In other words, again, acyclovir did not prevent PHN within one month after the onset of the rash. The results of a heterogeneity assessment showed no significant heterogeneity among these three studies (χ^2^ = 0.52, *p* = 0.77, *I*^2^ = 0%) (Figure 4c).

#### 3.10.4. Sensitivity Analysis

Overall, then, we found no significant heterogeneity in our meta-analysis of the effectiveness of acyclovir in relieving PHN within one month after the onset of the acute herpetic rash. We excluded two of the five studies from this analysis because of a high or an uncertain risk of bias [78,80] and conducted a sensitivity analysis on the other three, which had a low risk of bias [52,53,67]. The sensitivity analysis found no significant difference between the groups that did and did not receive acyclovir (RR = 1.12, 95%, CI: 0.99 to 1.28, *p* = 0.07). The results of a heterogeneity assessment also showed no significant heterogeneity among the studies (χ^2^ = 1.46, *p* = 0.48, *I*^2^ = 0%) (Figure 4d).

## 4. Discussion

### 4.1. Summary of the Main Results

We identified five randomized controlled studies that examined the treatment effect of various antiviral agents on PHN. Three of the studies compared the antiviral acyclovir with a placebo [53,67,77], and two compared acyclovir with a prodrugs, either famciclovir [80] or valaciclovir [52]. Overall, the five studies showed that acyclovir was no more effective in reducing the incidence of PHN within one month than a placebo or the other treatments (RR = 1.13, 95%, CI: 0.99 to 1.28, *p* = 0.06). Rather, the participants who received the prodrugs showed a significantly greater remission of PHN (RR = 0.86, 95% CI: 0.75 to 0.98, *p* = 0.03) compared with those who received acyclovir. In other words, these new antiviral agents were more effective in relieving PHN than acyclovir.

One possible explanation for this result is that acyclovir may simply be less effective than the new antiviral agents in treating PHN [52,80,84]. Our finding that the patients who received the prodrugs had significantly lower risks of PHN than those who received acyclovir (again, RR = 0.86, 95% CI: 0.75 to 0.98) is consistent with the findings of other recent studies [42,45,52,54,85]. In terms of the pharmacokinetic properties of these drugs, valaciclovir is better absorbed in the gastrointestinal tract and undergoes rapid and extensive first-pass metabolism [86]. Furthermore, valaciclovir appears to have significantly greater bioavailability than acyclovir (approximately threefold to fivefold) [87]. Like valaciclovir, famciclovir is rapidly metabolized in the intestine and liver into penciclovir, which provides prolonged antiviral activity [85]. Moreover, famciclovir also demonstrated better drug tolerance, a more favorable safety profile, and greater bioavailability than acyclovir [51,80]. Overall, then, new antiviral agents had better efficacy in relieving PHN than acyclovir. 

The difference in efficacy between the prodrugs and acyclovir may also contribute to patients’ non-adherence to treatment. As discussed, the former are more convenient than acyclovir in terms of being administered at lower unit dosages and frequencies. Dosage and frequency are key factors in non-adherence to medication, which a previous review study associated, unsurprisingly, with adverse treatment outcomes [62]. Simply put, the likelihood of patients completing a course of treatment may correlate inversely with the frequency and size of the doses administered, for which reason the prodrugs may have shown superior efficacy in relieving PHN compared to acyclovir. Conversely, non-adherence increases the likelihood of treatment failure and costs associated with medical conditions, increasing the burden on healthcare systems [88]. For the management of PHN, then, the prodrugs were significantly more cost-effective than acyclovir. 

We also found that the prodrugs may show their effects more quickly than acyclovir after the onset of the rash. Beutner et al. (1995) likewise [81] found that valaciclovir resolved the pain associated with HZ significantly more rapidly than acyclovir (HR =1.24, 95% CI 1.04 to 1.48, *p* = 0.01). The results of one double-blind RCT also indicated that patients treated with famciclovir decreased the duration of their PHN resolution by 10 days [53], though the evaluation and definition of PHN employed in the study adhered to no uniform guidelines. Other studies have defined PHN according to the time of onset; the definitions used in the five studies included in this meta-analysis ranged from 1 to 6 months. For the purpose of comparison, we extracted the data from these studies at the same time point, which was the least time after the onset of HZ (i.e., 1 month), to examine the efficacy of the treatments with the antiviral agents. A meta-analysis by Chen et al. (2014) found no significant variation in efficacy associated with variation in the period of treatment for PHN (at 1 month, RR = 0.99, 95% CI 0.83 to 1.17, *p* = 0.89; at 4 months, RR = 0.98, 95% CI 0.59 to 1.62, *p* = 0.93; at 6 months, RR = 1.08, 95% CI 0.61 to 1.91, *p* = 0.80). As mentioned, PHN may persist for months or years, [20,28,29] and previous research also highlighted the importance of beginning antiviral therapy for HZ early for reducing the incidence of PHN [89]. Accordingly, the prodrugs are likely to be most effective in relieving PHN when administered as early as possible in the course of HZ. Still, more evidence from further RCTs involving longer periods of observation is necessary to assess the effectiveness of the prodrugs in relieving PHN.

The incidence of PHN in the control groups in our meta-analysis ranged from 30% to 50%, proportions comparable to previous reports of the incidence of PHN in the general population, which ranged from 10% to 57.5% [51,90]. The incidence of PHN in the acyclovir groups in our meta-analysis ranged from 31% to 56%, figures also largely consistent with previous reports, which ranged from 10% to 34% [6,22,30,31,32,33,34,35]. The difference appears to be attributable to the various methodologies employed in the various studies. First and foremost, Surman et al. (1995) used the smallest sample (*n* = 20) among the five studies, acknowledged this as an important limitation of their study, and called for clinical trials with larger samples to explore further the association between acyclovir and PHN. These researchers’ finding of the highest incidence of PHN in the acyclovir group (56%) is consistent with the results of other previous researchers [51,90]. In addition, some investigators have proposed other new treatments for PHN that out-perform acyclovir [33,35]. 

While none of the five studies in our meta-analysis reported any serious adverse events, some participants did experience mild nausea, headaches, constipation, dyspepsia, and vomiting [52,53,67,78,80]. Surman et al. (1990) reported that one participant in their placebo group developed a rash but did not conduct a statistical analysis in this regard. Morton and Thomson (1989) reported one death each in their treatment and control groups, but neither was related to the intervention.

### 4.2. Overall Completeness and Applicability of the Evidence

Four of the five studies in our meta-analysis identified the endpoint of PHN clearly. The other, by Surman et al. (1990), identified PHN based on clinically significant reductions in pain. The fact that none of the participants in any of the five studies were immunocompromised limited the generalizability of our results since immunocompromised individuals are especially at risk of HZ and PHN, i.e., since we were unable to evaluate the effects of acyclovir on PHN for members of this population, the significance of our results for the immunocompromised can only be inferred.

### 4.3. Quality of the Evidence

Though all five of the studies in our meta-analysis used an RCT design, each had one or more risk of bias. Beutner et al. (1995), Surman et al. (1990), and Morton and Thomson (1989) enrolled participants who were receiving other treatments and, therefore, may have underestimated the effects of the intervention. Huff et al. (1988) conducted a blinding procedure but did not describe their method for allocation concealment. Gopal et al. (2013) did not mention the blinding procedure with respect to the participants, personnel, or outcome measurement, for which reason we rated the risk of bias in their study as high and the certainty as low, therefore precluding any robust conclusions regarding the efficacy of acyclovir in treating PHN.

### 4.4. Potential Biases in the Review Process

As mentioned, the definition of PHN was a major issue across the five studies. In general, the evaluation of PHN has been complicated by the lack of a universally accepted definition for the condition. Further studies could help to address this gap in the research by exploring the application of a global standard diagnosis of and definition for PHN, such as in the context of the International Statistical Classification of Diseases (ICD). Use of the ICD could ensure the semantic interoperability and reusability of recorded data for the various use cases and, therefore, precision in the identification of PHN. Additionally, reducing the risk of misclassifying PHN would help to clarify assessments of the efficacy of the various antiviral agents used to treat the condition.

Though we took every measure available to identify suitable studies that met our criteria in the biomedical and life sciences literature databases, our sample was relatively small. In part, this outcome was a consequence of the fact that we did not include studies published in languages other than English. The five studies were insufficient for us to employ funnel plots to assess the risk of publication bias, the presence of which can only be inferred.

### 4.5. Consistency with Previous Studies and Reviews

To the best of our knowledge, only one systematic review and meta-analysis evaluated the efficacy of acyclovir in treating PHN, with the researchers concluding that it is not, in fact, effective [45]. Similarly, in the current study, the patients who received acyclovir had no less risk of PHN than those in the control groups that received either a placebo or a prodrugs (RR = 1.13, 95%, CI: 0.99 to 1.28, *p* = 0.06). Chen et al. (2014) did not compare the efficacy of the prodrugs with that of acyclovir in relieving PHN because they were unable to identify enough eligible RCTs for such an analysis. However, our study included two studies that made this comparison [52,80]. The findings therein, as reviewed in the current study, thus help to fill the gap that Chen et al. identified, suggesting that the prodrugs are, indeed, significantly better than acyclovir in the management of PHN.

### 4.6. Implications for Practice and Research

The findings presented here demonstrating the efficacy of the newer antivirals in relieving PHN are also consistent with the evidence reported in previous studies that these agents can decrease the severity and/or reduce the duration of PHN [52,80]. Since our search of the database identified no relevant studies that included immunocompromised patients, though, further research is needed to assess the efficacy of these agents in such population. For example, another recent meta-analysis found that individuals with diabetes mellitus (DM) were at greater risk of HZ than general population (pooled relative risk [RR]: 1.38; 95% CI, 1.21–1.57) as well as a dose-response association between age and the risk of HZ within the DM population [91]. Similar results have also been reported in the adult population for individuals with diabetes, who experienced a higher adjusted risk of HZ (hazard ratio = 1.45; 95% CI: 1.43 to 1.46) and higher adjusted odds of persistent post-zoster pain (OR = 1.18, 95% CI: 1.13 to 1.24) [92]. Studies of the use of the prodrugs to treat the members of these populations are needed that are well-designed, randomized, and involve relatively large sample sizes in order to explore these issues further.

### 4.7. Limitations

The limitations of this meta-analysis also need to be acknowledged in the interpretation of the overall findings. Above all, the search strategy excluded from the results studies that were not both in English and published. However, it would be difficult to assess the content and quality of studies that do not meet these criteria. While the five studies that we analyzed employed a randomized, double-blind design, we considered the influence of this limitation negligible. A further limitation was the small sample size, which resulted in the low moderator power of the RCTs in our analysis. To alleviate this limitation, future studies should involve larger RCTs. Moreover, some potential confounding factors, such as concomitant medications and comorbidities among patients, may have had a direct impact on the outcome measure. It is unknown whether these potential confounders were considered in the analysis conducted by the researchers responsible for the five studies analyzed here, though such biases could have a tremendous impact on the findings that they reported. Lastly, the characteristics of the participants, and, in particular, their status as immunocompetent, may limit the generalizability of this meta-analysis since immunocompromised individuals are at especially high risk of HZ and PHN. To the best of our knowledge, no study has yet focused on the treatment of PHN with prodrugs in immunocompromised patients, so there is a need for future research of this sort with a retrospective design and drawing on a national database.

## 5. Conclusions

Our meta-analysis found that the prodrugs valaciclovir and famciclovir were effective in relieving PHN among HZ patients. However, the evidence that we analyzed was insufficient to determine whether acyclovir can have this effect. We did find that the prodrugs were more effective than acyclovir for mitigating PHN. These agents have been in use for years, but relatively few recent RCTs have focused on them in this regard. We conclude that their efficacy in general and for especially susceptible populations such as the immunocompromised and those with DM in particular merits further investigation. More large-scale, multicenter RCTs with a wide range of participants are needed to verify and build on our findings.

## Data Availability

The data used in this study are available on reasonable request from the corresponding author (S.-L.T.).

## Supplementary Materials

The following supporting information can be downloaded at: https://www.mdpi.com/article/10.3390/healthcare10071181/s1, Supplement Table S1.: Search Strategy.; Supplement Table S2: The summary of including studies characteristics.; Supplement Table S3: The summary of including studies characteristics and risk of bias.
