# Supplementary material for: Comparing Prodrugs with Acyclovir for Treating Postherpetic Neuralgia among Herpes Zoster Patients: A Systematic Review and Meta-Analysis
Source: Healthcare (Basel). 2022 Jun 24;10(7):1181. doi: 10.3390/healthcare10071181 (PMC9322848; doi:10.3390/healthcare10071181)
Supplement: Supplementary file 1 [file healthcare-10-01181-s001.zip › healthcare-1766158-supplementary.pdf]

Supplementary Table S1. Search Strategy.

PubMed	
Steps	Number of studies
#1 "Herpes Zoster"[Mesh]	12459
#2 Herpes zoster [Title/Abstract]	10555
#3 "Acyclovir"[Mesh]	14783
#4 Acyclovir[Title/Abstract]	8491
#5 "Neuralgia, Postherpetic"[Mesh]	1221
#6 Postherpetic neuralgia[Title/Abstract]	2167
#7 #1 OR #2	15810
#8 #3 OR #4	17976
#9 #5 OR #6	2614
#10 #7 AND #8 AND #9	182
#11 limited to Clinical Trial	39
#12 limited to Clinical Trial, Randomized Controlled Trial	39
#13 limited to Clinical Trial, Meta-Analysis, Randomized Controlled Trial	45
Cochrane Central Register of Controlled Trials	
Steps	Number of studies
#1 (herpes zoster):ti,ab,kw (Word variations have been searched)	2291
#2 MeSH descriptor: [Herpes Zoster] explode all trees	471
#4 acyclovir	1464
#5 MeSH descriptor: [Acyclovir] explode all trees	1263
#6 Postherpetic neuralgia	1140
#7 MeSH descriptor: [Neuralgia, Postherpetic] explode all trees	283
#8 #1 OR #2	2291
#9 #3 OR #4	1819
#10 #5 OR #6	1140
#11 #7 AND #8 AND #9	5
Medline	
Steps	Number of studies
#1 herpes zoster.mp. or exp herpes zoster/ [mp=title, abstract, original title, name of substance word, subject heading word, floating sub-heading word, keyword heading word, organism supplementary concept word,	14688

protocol supplementary concept word, rare disease supplementary concept word, unique identifier, synonyms]	
#2 limit 1 to abstracts	8633
#3 acyclovir.mp. or exp Acyclovir/ [mp=title, abstract, original title, name of substance word, subject heading word, floating sub-heading word, keyword heading word, organism supplementary concept word, protocol supplementary concept word, rare disease supplementary concept word, unique identifier, synonyms]	17187
#4 limit 3 to abstracts	13537
#5 Postherpetic neuralgia.mp. or exp Neuralgia, Postherpetic/ [mp=title, abstract, original title, name of substance word, subject heading word, floating sub-heading word, keyword heading word, organism supplementary concept word, protocol supplementary concept word, rare disease supplementary concept word, unique identifier, synonyms]	2318
#6 limit 5 to abstracts	1967
#7 #2 AND #4 AND #6	151
Embase	
#1 'herpes zoster'/exp OR 'varicella zoster virus infection':ti,ab OR 'varicellovirus infection':ti,ab OR 'disseminated herpes zoster':ti,ab OR 'herpes zoster':ti,ab OR 'herpes zoster infection':ti,ab OR 'herpes zoster neuralgia':ti,ab OR 'herpes zoster paralysis':ti,ab OR 'shingles':ti,ab OR 'varicella zoster infection':ti,ab OR 'zoster':ti,ab OR 'zoster, herpes':ti,ab	38601
#2 'aciclovir'/exp OR '1, 9 dihydro 2 amino 9 [(2 hydroxyethoxy) methyl] 6 purinone':ti,ab OR '1, 9 dihydro 2 amino 9 [(2 hydroxyethoxy) methyl] 6h purin 6 one':ti,ab OR '2 amino 1, 9 dihydro 9 [(2 hydroxyethoxy) methyl] 6 purinone':ti,ab OR '2 amino 1, 9 dihydro 9 [(2 hydroxyethoxy) methyl] 6h purin 6 one':ti,ab OR '2 amino 6 hydroxy 9 (2 hydroxyethoxymethyl) purine':ti,ab OR '9 (2 hydroxyethoxymethyl) guanine':ti,ab OR '9 [(2	57531

hydroxyethoxy) methyl] guanine':ti,ab OR 'acerpes':ti,ab OR 'acevir':ti,ab OR 'acevirex':ti,ab OR 'acg':ti,ab OR 'acic':ti,ab OR 'acic creme':ti,ab OR 'aciclin':ti,ab OR 'aciclinlabiale':ti,ab OR 'acicloftal':ti,ab OR 'aciclor':ti,ab OR 'aciclosina':ti,ab OR 'aciclostad':ti,ab OR 'aciclovir':ti,ab OR 'aciclovir hydrochloride':ti,ab OR 'aciclovir sodium':ti,ab OR 'aciclovir-bc iv':ti,ab OR 'acihexal':ti,ab OR 'acilax cream':ti,ab OR 'acitop':ti,ab OR 'acivir cream':ti,ab OR 'acivir eye':ti,ab OR 'acivision':ti,ab OR 'aclova':ti,ab OR 'aclovir':ti,ab OR 'aclovirax':ti,ab OR 'activir':ti,ab OR 'acv':ti,ab OR 'acy':ti,ab OR 'acyclo-v':ti,ab OR 'acycloguanosine':ti,ab OR 'acyclovidar':ti,ab OR 'acyclovir':ti,ab OR 'acyclovir in sodium chloride 0.9% preservative free':ti,ab OR 'acyclovir sodium':ti,ab OR 'acylene':ti,ab OR 'acyron':ti,ab OR 'acyrova':ti,ab OR 'acyvir':ti,ab OR 'alovex':ti,ab OR 'alovexlabiale':ti,ab OR 'amodivyr':ti,ab OR 'antiviral cold sore cream (aciclovir)':ti,ab OR 'antix':ti,ab OR 'apicol':ti,ab OR 'avaclyr':ti,ab OR 'avirax':ti,ab OR 'avix':ti,ab OR 'avorax':ti,ab OR 'avorax cream':ti,ab OR 'axoviral':ti,ab OR 'azovir':ti,ab OR 'bearax':ti,ab OR 'bel labial':ti,ab OR 'bw 248u':ti,ab OR 'bw248u':ti,ab OR 'cicloferon':ti,ab OR 'cicloviral':ti,ab OR 'cicloviran':ti,ab OR 'clovicin':ti,ab OR 'clovir':ti,ab OR 'cloviran':ti,ab OR 'cold sore cream (aciclovir)':ti,ab OR 'colsor':ti,ab OR 'contra (drug)':ti,ab OR 'cusiviral':ti,ab OR 'cyclivex':ti,ab OR 'cyclomed':ti,ab OR 'cyclorax':ti,ab OR 'cyclostad':ti,ab OR 'cyclovir':ti,ab OR 'cycloviran':ti,ab OR 'cyllanvir':ti,ab OR 'danovir':ti,ab OR

'deherp':ti,ab OR 'dravyr':ti,ab OR
'dumophar':ti,ab OR 'duvimex':ti,ab OR
'eduvir':ti,ab OR 'efriviral':ti,ab OR
'efrivirallabiale':ti,ab OR 'entir':ti,ab OR
'erlvirax':ti,ab OR 'eurovir':ti,ab OR
'exavir':ti,ab OR 'expit':ti,ab OR 'genvir':ti,ab
OR 'glenlip':ti,ab OR 'guanine, 9 (2
hydroxyethoxymethyl)':ti,ab OR 'hermix
sofex':ti,ab OR 'herpefug':ti,ab OR
'herpetad':ti,ab OR 'herpex':ti,ab OR
'herpofug':ti,ab OR 'herpoviric':ti,ab OR
'herpoviric rp creme':ti,ab OR 'hevipoint':ti,ab
OR 'inmerax':ti,ab OR 'innovirax':ti,ab OR
'isavir':ti,ab OR 'juviral':ti,ab OR
'labiriad':ti,ab OR 'laciken':ti,ab OR
'leramex':ti,ab OR 'lermex':ti,ab OR
'lesaclor':ti,ab OR 'libravir':ti,ab OR
'lipovir':ti,ab OR 'lisovyr':ti,ab OR
'lovir':ti,ab OR 'lovire':ti,ab OR 'maclov':ti,ab
OR 'marvir':ti,ab OR 'matrovir':ti,ab OR
'maynor':ti,ab OR 'medovir':ti,ab OR
'norum':ti,ab OR 'olvit':ti,ab OR 'oppvir':ti,ab
OR 'opthavir':ti,ab OR 'poviral':ti,ab OR
'proviral':ti,ab OR 'qualiclovir':ti,ab OR
'quavir':ti,ab OR 'ranvir':ti,ab OR
'raxclo':ti,ab OR 'sitavig':ti,ab OR
'supra-vir':ti,ab OR 'supraviran':ti,ab OR
'supraviran creme':ti,ab OR 'syntovir':ti,ab OR
'tuclor':ti,ab OR 'vacrax':ti,ab OR
'vacrovir':ti,ab OR 'vicorax':ti,ab OR
'viraban':ti,ab OR 'viralex':ti,ab OR
'viralex-ds':ti,ab OR 'viranti':ti,ab OR
'viratop':ti,ab OR 'virax':ti,ab OR
'vircella':ti,ab OR 'virest':ti,ab OR
'virex':ti,ab OR 'virless':ti,ab OR
'viroclear':ti,ab OR 'virogon':ti,ab OR
'virolan':ti,ab OR 'virolex':ti,ab OR
'viromed':ti,ab OR 'vironida':ti,ab OR

'viropump':ti,ab OR 'viruderm':ti,ab OR 'virules':ti,ab OR 'virupos':ti,ab OR 'virupos eye oint':ti,ab OR 'viruseen':ti,ab OR 'vivir':ti,ab OR 'voraclor':ti,ab OR 'warviron':ti,ab OR 'wellcome 248 u':ti,ab OR 'zaclovir':ti,ab OR 'zetavir':ti,ab OR 'zeven cream':ti,ab OR 'zevin':ti,ab OR 'zodiac (drug)':ti,ab OR 'zoral':ti,ab OR 'zoral cream':ti,ab OR 'zorax':ti,ab OR 'zorel':ti,ab OR 'zoter':ti,ab OR 'zovir':ti,ab OR 'zovirax':ti,ab OR 'zovirax topical':ti,ab OR 'zoviraxlabiale':ti,ab OR 'zoylex':ti,ab OR 'zumasid':ti,ab OR 'zyclir':ti,ab OR 'zyvir':ti,ab	
#3 neuralgia':ti,ab OR 'neuralgia, postherpetic':ti,ab OR 'postherpetic neuralgia':ti,ab OR 'postherpetic pain':ti,ab	6969
#4 #1 AND #2 AND #3	1041
#5 limited to [meta analysis] OR [controlled clinical trial OR [randomized controlled trial]/lim)	101

Supplementary Table S2. The summary of including studies characteristics.

		participants				
Author	Study design	No. of patients with PHN in acyclovir	No. of patients with PHN in control	Mean ages	Intervention	Outcome of interest
(Beutner et al., 1995)	A randomized, double-blind, study in multicenter	214/376	188/384	Acyclovir group:68 Control group:69	Participants received acyclovir 800 mg 5 times daily for 7 days were identified as acyclovir group. Participants received valaciclovir 1,000 mg 3 times daily for 7 days were identified as control group.	The presence of PHN persisting for more than 30 days.
(Gopal et al., 2013)	A comparative randomized	3/50	2/50	Acyclovir group:52.5	Participants received	The presence pain after initial

	clinical study in in one medical sciences hospital.			Control group:49.64	acyclovir 800 mg 5 times daily for 7 days were identified as acyclovir group. Participants received famciclovir 250 mg 3 times daily for 7 days were identified as control group.	treatment for 6 weeks.
(Huff, 1988)	A randomized, double-blind, placebo control study in multicenter	29/93	28/94	Acyclovir group:49 Control group:50	Participants received two doses of acyclovir (800 mg, 5 times per day) for 10 days were identified as acyclovir group. Participants	The presence of PHN for 6 months post-treatment

					received placebo were identified as control group.	
(Morton & Thomson, 1989)	A randomized, double-blind, placebo control study in one center	13/40	17/43	Acyclovir group:50.8 Control group:52.5	Participant received acyclovir (800 mg, 5 times per day) for 7 days were identified as acyclovir group. Participants received placebo for 7 days were identified as control group.	The presence of PHN 1 month after the onset of HZ.
(Surman et al., 1990)	A randomized, double-blind, placebo control study	4/9	4/8	Acyclovir group:71.6 Control group:68.6	Participant received acyclovir (800 mg po every four hours, during waking	The presence of pain 1 month after initial treatment.

					hours) for 12 weeks were identified as acyclovir group. Participants received placebo tablets were identified as control group.	

Supplementary Table S3. The summary of including studies characteristics and risk of bias.

Beutner et al., 1995	
Methods	Multicenter, randomized, three-arm, double-blind, double-dummy study.
Participants	Patients 50 years of age or older with clinically diagnosed, localized herpes zoster presenting within 72 hours after the onset of rash were enrolled in this study.<br><br>Pregnant, nursing, and sexually active women of childbearing potential were excluded.<br><br>Patients treated with other antiviral medications, immunomodulating agents, or capsaicin within the previous 4 weeks or those receiving probenecid or tricyclic antidepressant medications at the time of entry were excluded.<br><br>Patients with congenital, acquired, or steroid-induced immunodeficiency, including malignancy; impaired renal function (estimated creatinine clearance of ≤35 ml/min or serum creatinine of ≥120 μmol/liter) or impaired hepatic function (either alanine aminotransferase or aspartate aminotransferase level more than threefold the upper limit of normal at the time of entry); or gastrointestinal dysfunction that might interfere with drug absorption were also excluded.<br><br>The mean age of group 1 (acyclovir), group 2 (valaciclovir for 7 days), and group 3 (valaciclovir for 14 days) was 68, 69, and 68 respectively.
Interventions	Patients receive treatment with valaciclovir at 1,000 mg three times daily for 7 days, valaciclovir at 1,000 mg three times daily for 14 days, or acyclovir at 800 mg five times daily for 7 days. All patients received

	study medication for 14 days.	
Outcomes	The presence of PHN persisting for 14 days or more than 30 days.	
Notes	The use of medications to control pain was permitted, which might lead to potential difference between groups.<br><br>A total of 1,141 patients were enrolled 107 study centers in 13 countries.	
Risk of bias		
Bias	Authors' judgment	Support for judgement
Random sequence generation (selection bias)	Low risk	Participants were randomized according to a computer-generated code.
Allocation concealment (selection bias)	Low risk	The computer-generated randomization code was conducted to allocate participants to groups.
Blinding of participants and personnel (performance bias)	Low risk	The author conducted a double-blind design.
Blinding of outcome assessment (detection bias)	Low risk	The author conducted a double-blind design, and the outcome were reported by participants themselves.
Incomplete outcome data (attrition bias)	Low risk	The author had reported the incomplete data, but the exact number of each group was not clear. There were 195 patients who did not complete the study: protocol violation (n=71 patients), adverse experiences (n=38), withdrawal of consent (n=30), and loss to follow-up (n=24). However, these

		reasons were similar distributed across groups. Authors performed an intent-to-treat analysis.
Selective reporting (reporting bias)	Low risk	The result of outcome was reported in the manuscript.
Other bias	Unclear risk	The study was predominantly outpatient based, hence, all participants received treatment, which might underestimate the effects of intervention.
Gopal et al., 2013		
Methods	A comparative randomized clinical study	
Participants	Immunocompetent patients over the age of 40 years with uncomplicated herpes zoster, characterized by localized, cutaneous lesions (papules or vesicles) presenting within 72 hours of the onset of the rash were included.<br>Patients were excluded by as follow:<br>1.Patients with complications of herpes zoster, including ocular involvement, severe disseminated infection, motor neuropathies, encephalitis or cerebrovascular complications.<br>2.Concurrent malignancy receiving chemotherapy<br>3.HIV sero-positive.<br>The mean age of group 1 (acyclovir) and group 2 (famciclovir) was 52.5 (SD=10.38) and 49.64 (SD=8.30).	
Interventions	Patients received 800mg of acyclovir 5 times daily or famciclovir 750mg thrice daily for a period of 7 days.	
Outcomes	The presence pain and healing of the cutaneous lesions on day 7 after initiation of therapy and every week thereafter, for a period of 6 weeks.	

Notes	This study did not include a placebo control and the definition of PHN was not clear.	
Risk of bias		
Bias	Authors' judgment	Support for judgement
Random sequence generation (selection bias)	Unclear risk	The method of random sequence generation was unclear.
Allocation concealment (selection bias)	Unclear risk	Participants were randomized 1:1 ratio to acyclovir group and famciclovir group, but authors did not describe the details of randomization.
Blinding of participants and personnel (performance bias)	High risk	The author did not mention the blinding procedure in the method section.
Blinding of outcome assessment (detection bias)	High risk	The author did not mention the blinding procedure in the method section.
Incomplete outcome data (attrition bias)	Low risk	The data showed that both groups did not have attrition data. However, the author did not mention the information about incomplete outcome data in the method section.
Selective reporting (reporting bias)	Low risk	The result of outcome was reported in the manuscript.
Other bias	Unclear risk	This study did not include a placebo control, which indicated that the effect of both the drugs on PHN would not be drawn clearly.
Huff, 1988		

Methods	A randomized double-blind study in multicenter.	
Participants	There were 187 patients with herpes zoster were included in this study and 52.41% (98/187) participants were 50 years old or older.<br>Patients who were less 18 years old, pregnant or nursing, immunosuppressed, hepatic or renal dysfunction, or received any antiviral therapy within 10 days were excluded.	
Interventions	Participant received two doses of acyclovir (400 mg, 5 times per day and 800 mg, 5 times per day). However, authors did not report the incidence of PHN among lose dose group and we extracted the high dose group only (n=93) and the placebo group (n=94).	
Outcomes	The presence of PHN for 6 months post-treatment.	
Notes	This study was conducted in 6 medical centers in USA.	
Risk of bias		
Bias	Authors' judgment	Support for judgement
Random sequence generation (selection bias)	Low risk	The random sequence was conducted by a computer.
Allocation concealment (selection bias)	Unclear risk	The author did not describe the method of allocation clearly.
Blinding of participants and personnel (performance bias)	Low risk	Participants were assigned in a double-blind sequence.
Blinding of outcome assessment (detection bias)	Low risk	The author conducted a double-blind design, and the outcome were reported by participants themselves.
Incomplete outcome data (attrition bias)	Low risk	There was total 36 participants (19%) lost to follow-up. Acyclovir group: 23% (21/93); Placebo group:

		16% (15/94).<br>The author did not perform the intent-to-treat analysis.
Selective reporting (reporting bias)	Low risk	The result of outcome was reported in the manuscript.
Other bias	Low risk	There would be no other potential bias.
Morton & Thomson, 1989		
Methods	A randomized double-blind study in one center.	
Participants	There were 83 immunocompetent people with herpes zoster rash were enrolled in this study. Patients who were presentation more than 72 hours after onset of rash; presence of known renal insufficiency; and pregnancy were excluded.<br>Forty patients aged 16 years or over, presenting to their general practitioners within 3 days of rash onset, received acyclovir, while 43 patients received placebo.	
Interventions	Participant received acyclovir (800 mg, 5 times per day) and placebo for 7 days.	
Outcomes	PHN 1 month after the onset of the acute herpetic rash.	
Notes	This study was conducted in the Wellcome Research Laboratories, Beckenham, UK.	
Risk of bias		
Bias	Authors' judgment	Support for judgement
Random sequence generation (selection bias)	Low risk	The participant was assigned with the numbered bottles that were generated and randomized by the center.
Allocation concealment	Low risk	All procedures were randomized by the Wellcome

(selection bias)		Research Laboratories, Beckenham, UK.
Blinding of participants and personnel (performance bias)	Low risk	Investigators, research nurse, general partitioners, and patients were all blinded.
Blinding of outcome assessment (detection bias)	Low risk	Participants were blind to their treatment status and the outcome were reported by participants themselves.
Incomplete outcome data (attrition bias)	Low risk	The author reported the incomplete data clearly. One died in both group and 1 withdrew from study during follow-up.<br>The author did not perform the intent-to-treat analysis.
Selective reporting (reporting bias)	Low risk	The result of outcome was reported in the manuscript.
Other bias	Unclear risk	The study allowed participants receive other treatment, which could potentially conduct a difference between two groups.
Surman et al., 1990		
Methods	A double-blind, placebo control study.	
Participants	There were 21 participants were enrolled in the study.<br>Participants who were acyclovir allergy, renal impairment, dementia, psychosis, and who received other antiviral agents were excluded.<br>The age range was 47 to 82 years (mean: 71.6 years) for those receiving acyclovir, and 56 to 81 years (mean:	

	68.6 years) for the placebo group.	
Interventions	One group received 12 weeks of acyclovir (800 mg po every four hours, during waking hours), while the second group received placebo tablets identical in appearance.	
Outcomes	Pain perception from 2 week to 6 months. For the comparison, we extracted the acyclovir group (n=9) and the placebo group (n=8) at after 1 month as participants received interventions.	
Notes	Study patients were recruited through advertisements in newspapers and medical journals, and through letters to subspecialty departments of neurology, dennatology, and infectious disease at New England area hospitals.	
Risk of bias		
Bias	Authors' judgment	Support for judgement
Random sequence generation (selection bias)	Unclear risk	Participants were randomized between two groups (acyclovir and placebo) on a double-blind basis, but authors did not describe the details of randomization.
Allocation concealment (selection bias)	Low risk	The author indicated that the participants were randomized into two groups on a double-blind basis.
Blinding of participants and personnel (performance bias)	Low risk	The author performed a double-blind design.
Blinding of outcome assessment (detection bias)	Low risk	The author performed a double-blind design and participants reported the outcome by themselves.
Incomplete outcome data (attrition bias)	Low risk	The author reported the incomplete data in each duration follow-ups.<br>The author did not perform the intent-to-treat

		analysis.
Selective reporting (reporting bias)	Low risk	The result of outcomes was reported in the manuscript.
Other bias	Unclear risk	Study participants were allowed to continue any current medications they were using for pain control, which might lead to the difference between groups. However, the result of this study indicated that the patterns of current pain-medication use were similar for the two groups.
